# ﻿Invalid lectotypification for *Synodontisvictoriae* Boulenger, 1906 (Siluriformes, Mochokidae) by Poll (1971), and the designation of a new lectotype

**DOI:** 10.3897/zookeys.1183.111868

**Published:** 2023-10-27

**Authors:** Gernot K. Englmaier, Rupert A. Collins

**Affiliations:** 1 Institute of Vertebrate Biology, Czech Academy of Sciences, Květná 8, 60365 Brno, Czech Republic Institute of Vertebrate Biology, Czech Academy of Sciences Brno Czech Republic; 2 Natural History Museum Vienna, Burgring 7, A-1010 Vienna, Austria Natural History Museum Vienna Vienna Austria; 3 Department of Life Sciences, Natural History Museum, Cromwell Road, London, SW7 5BD, UK Natural History Museum London United Kingdom

**Keywords:** Catfish, East Africa, freshwater fish, ICZN, taxonomy

## Abstract

The lectotype and paralectotype of *Synodontisvictoriae* Boulenger, 1906, designated by Poll (1971), were examined. Inconsistencies between data presented for the designated lectotype and the illustrated individual raise the question of whether lectotypification by Poll is valid. This case is not formally regulated by the International Code of Zoological Nomenclature, but based on Article 74.5, the lectotypification for *S.victoriae* should be considered invalid because it cannot unambiguously indicate a single name-bearing specimen. Thus, we designate a new lectotype for *S.victoriae* (BMNH 1906.5.30.191, Entebbe, standard length 188.2 mm) out of two syntypes and provide illustrations and new morphometric and meristic data for both specimens.

## ﻿Introduction

**Figure 1.**
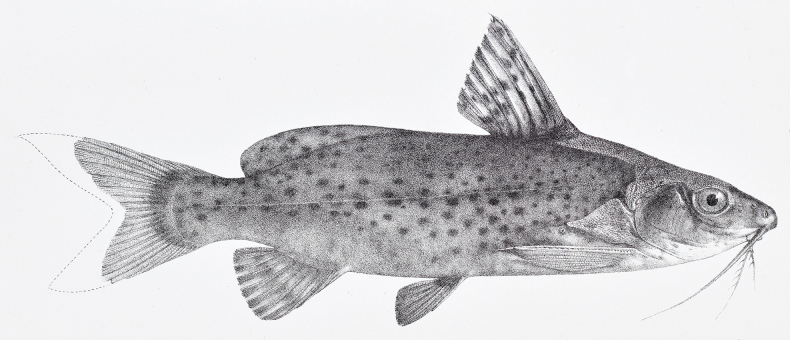
First illustration of *Synodontisvictoriae* published in "*The Fishes of the Nile*" (modified from Boulenger 1907: pl. LXVII) representing the specimen from Entebbe (BMNH 1906.5.30.191) and not from Buganga (BMNH 1906.5.30.190) as stated by Boulenger (1907: xiii). A reproduction of this right lateral view was used by Poll (1971: 121, fig. 50).

Based on an extensive collection of fishes from Lake Victoria made by Edward J.E. Degen in 1905, Boulenger (1906) described 26 new species, including *Synodontisvictoriae* Boulenger, 1906. The original description of this species was based on two syntypes from the north-western part of Lake Victoria: one from Entebbe (collected on 1 October 1905; standard length (SL) 192 mm) and a second from Buganga (collected on 15 November 1905; SL 225 mm) (Boulenger 1907). Additional descriptive data were provided in Boulenger’s "*The Fishes of the Nile*", where the first illustration of *S.victoriae* appeared (Boulenger 1907: pl. LXVII) (Fig. 1). In a comprehensive revision of the genus, Poll (1971) designated the larger of the two syntypes as the lectotype (BMNH 1906.5.30.190, from Buganga).

After careful examination of the two type specimens, we found inconsistencies in both the information presented by Poll (1971) and the labels on and in the jars containing the specimens. This ambiguity regarding the identity of the name-bearing specimen questions the validity of the lectotype designation for *S.victoriae* by Poll (1971). Here, we discuss this case in light of Article 74 of the International Code of Zoological Nomenclature (ICZN 1999), designate a new lectotype, and provide illustrations and new morphometric and meristic data for both type specimens.

## ﻿Material and methods

The types of *Synodontisvictoriae* were examined, and 36 morphometric measurements and 20 meristic characters, including four axial skeleton counts (from radiographs), were taken. Measurements were made point-to-point using callipers to the nearest 0.1 mm. Most measurements (21) follow Skelton and White (1990), seven are summarised in Englmaier et al. (2020) (body depth at dorsal fin origin, pectoral–pelvic distance, pelvic–anal distance, anal-fin depth, pectoral-fin length, pelvic-fin length, and minimum caudal-peduncle depth). Eight additional measurements were conducted as follows: body depth at anal fin refers to the greatest vertical distance (including the height of the adipose fin) at origin and insertion of the anal fin; dorsal fin to caudal peduncle was measured from the insertion of the dorsal fin to the posterior margin of the last complex centrum at midline; head depth and head width at posterior eye margin were measured as greatest vertical and lateral distances; maximum cranium width was measured between lateral margins of pterotics; head length was measured from the tip of snout to the posterior margin of the soft gill cover; and width of mandibular teeth row refers to a maximum distance between the outermost visible replacement teeth. Counts of external meristic traits and axial skeleton elements follow Skelton and White (1990); caudal-fin ray counts as described in McDowall (2001). The posterior two branched rays in the anal fin, located on the last complex proximal pterygiophore of the fin, were counted as two. Vertebral counts were made from radiographs and include six Weberian vertebrae (the 6^th^ centrum already with ribs) and a single count for the last complex centrum (Skelton and White 1990). The first true caudal vertebra is considered as a vertebra with “fully developed haemal spine” being similar in length as the haemal spine of the vertebra behind it.

## ﻿Taxonomic remarks

**Figure 2.**
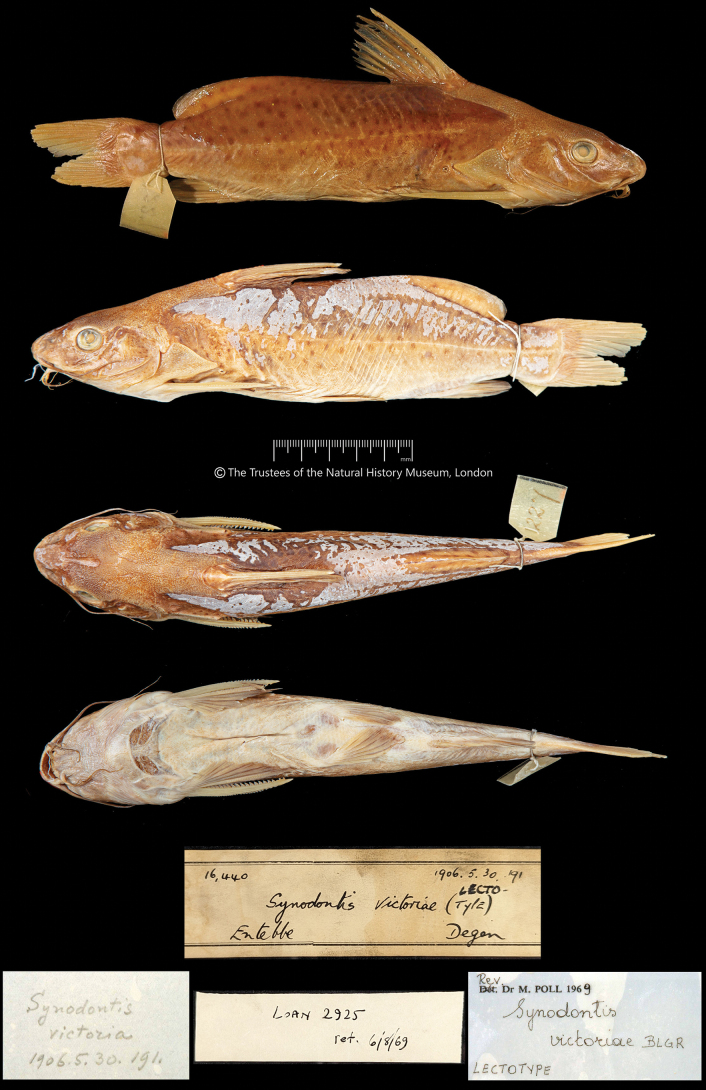
*Synodontisvictoriae* BMNH 1906.5.30.191, lectotype, 188.2 mm SL, Entebbe, Lake Victoria, Uganda. The Trustees of the Natural History Museum, London.

**Figure 3.**
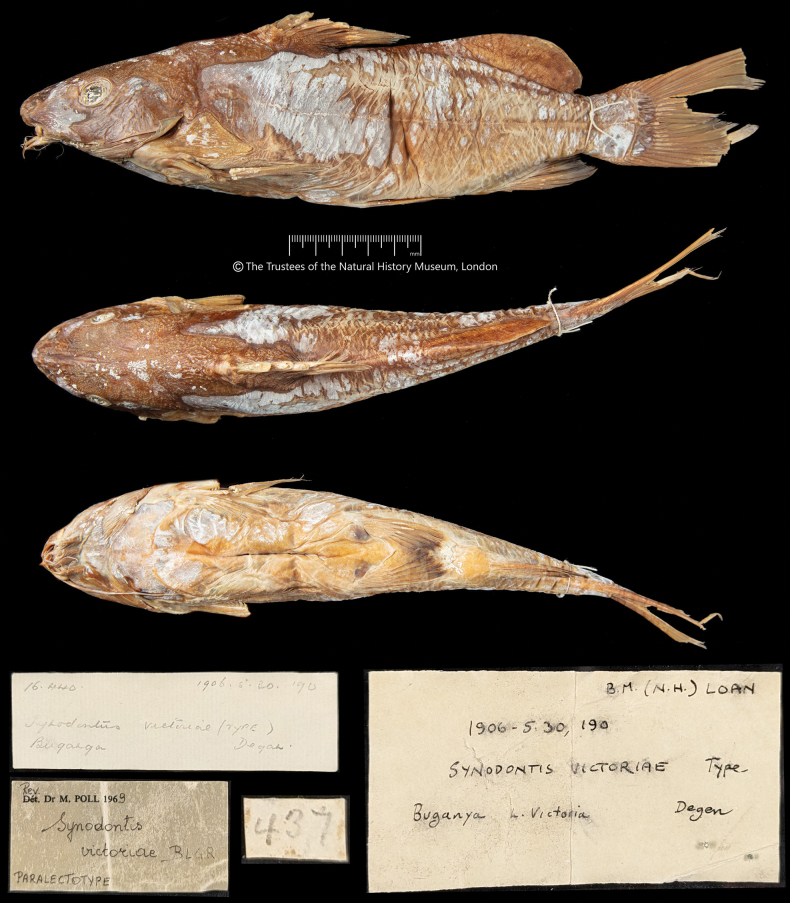
*Synodontisvictoriae* BMNH 1906.5.30.190, paralectotype, 225.0 mm SL, Buganga, Lake Victoria, Uganda. The Trustees of the Natural History Museum, London.

The two types of *Synodontisvictoriae* differ considerably in state of preservation and can therefore be easily distinguished (Figs 2, 3). The smaller specimen from Entebbe has distinct body markings, and the dorsal and pectoral fin spines are entirely preserved, whereas in the larger specimen from Buganga, the body markings are indistinct and the spines in the dorsal and pectoral fins are broken. This must have already been the case when Boulenger examined the material, because data for the dorsal and pectoral spines are missing for the specimen from Buganga (Boulenger 1907: 363).

Inconsistencies regarding the lectotype designation by Poll (1971), as well as with the internal and external jar labels, were noted as follows:

1. While Poll (1971) indicated the syntype from Buganga as lectotype by collection number, locality, and morphology, the illustration (Poll 1971: 121, fig. 50) with the legend that reads “ *Synodontisvictoriae* BOULENGER, lectotype, 290 mm, Buganga, lake Victoria (BRIT. MUS. no 1906.5.30.190). Partim G. A. BOULENGER, 1907, Fishes of the Nile, pl. LXVII” actually represents the syntype from Entebbe (BMNH 1906.5.30.191) (Fig. 1). This reproduction from Boulenger (1907) is the right lateral view of the specimen. In both publications, Boulenger (1907: xiii) and Poll (1971: 121), the illustrated syntype is erroneously referred to as originating from Buganga. The two additional illustrations provided by Poll (Poll 1971: 121, fig. 50; dorsal and ventral view) show a specimen with entire pectoral spines and thus cannot refer to BMNH 1906.5.30.190, a specimen where pectoral spines are broken.
2. The labels, on and in both jars and signed by Poll, however, identify the specimen from Entebbe as the lectotype and the specimen from Buganga as the paralectotype (Figs 2, 3), in contrast to (1).

This situation does not allow to unequivocally identify a unique name-bearing type in *S.victoriae*, presenting a nomenclatural problem when lectotype designation cannot be unambiguously traced back to a single specimen. Article 74.5 of the ICZN (1999) states that “In a lectotype designation made before 2000, either the term ‘lectotype’, or an exact translation or equivalent expression (e.g. ‘the type’), must have been used or the author must have unambiguously selected a particular syntype to act as the unique name-bearing type of the taxon”. This implies that a single specimen is chosen “… to become the unique bearer of the name of a nominal species-group taxon ...”. (Article 74.1, ICZN 1999), and that this specimen can be unambiguously traced back from the context of the original work. Although Poll (1971) assigned the term “lectotype” to a specific syntype, recognised by collection number, locality, and morphology, the illustration of the lectotype refers to a different specimen, resulting in a composite description of two syntypes. Additional ambiguity is introduced by the jar labels added by Poll, which would identify a different lectotype than designated by description. Articles 72 and 74 of the ICZN (1999) provide specific recommendations regarding labelling of type specimens: Recommendation 72D “… Holotypes, syntypes, lectotypes and neotypes should be labelled in a way that will unmistakably denote their status” and Recommendation 74E “… An author who designates a lectotype should clearly label other former syntypes as ‘paralectotypes’ …”. These recommendations are also stated in the 2^nd^ edition of the ICZN (1964), valid at the time Poll designated the lectotype of *S.victoriae*, in 72B and 74E, respectively. The ICZN also provides a recommendation regarding the selection of a lectotype if a syntype has previously been illustrated, stating that: “… A zoologist should choose as lectotype a syntype of which a figure has been published, if such exists” (Recommendation 74B, ICZN 1964; see also Recommendation 74B, ICZN 1999). This could probably explain the original intention of Poll to designate the smaller syntype from Entebbe as the lectotype during his visit to (and loans from) the British Museum (Natural History) in 1969–1970, as this specimen was illustrated by Boulenger (1907).

From the discussion above, we conclude that the lectotypification for *S.victoriae* by Poll (1971) should be considered invalid because it cannot be unambiguously traced back to a single name-bearing specimen. We herewith designate a new lectotype, out of the two syntypes, as follows.

### ﻿ Synodontis victoriae

Taxon classificationAnimaliaSiluriformesMochokidae

Boulenger, 1906: 438

74D9F25E-1496-5947-A627-6509E2DC4E9F

#### Type materials.

***Lectotype*** (hereby designated): BMNH 1906.5.30.191, Entebbe, 188.2 mm SL, coll. E. Degen.

***Paralectotype***: BMNH 1906.5.30.190, Buganga, 225.0 mm SL, coll. E. Degen.

#### Notes.

**Figure 4.**
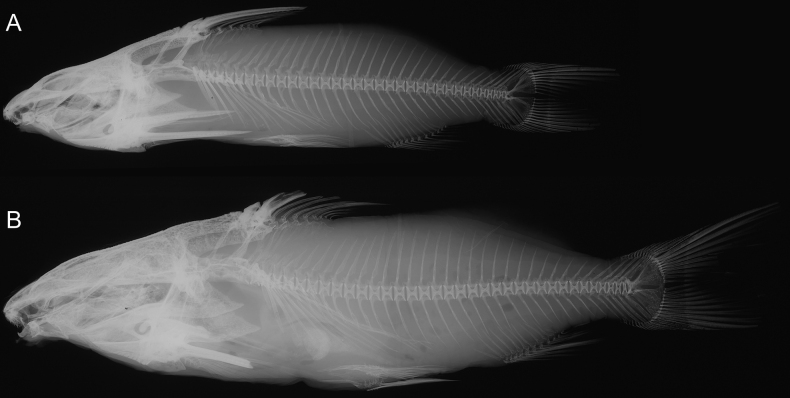
Axial skeletons in *Synodontisvictoriae***A** BMNH 1906.5.30.191, lectotype **B** BMNH 1906.5.30.190, paralectotype.

In Figs 2–4 we provide illustrations and radiographs (axial skeletons) of both the lectotype and paralectotype of *S.victoriae*; new morphometric and meristic data of the two specimens are given in Table 1.

**Table 1.** Morphometric measurements and meristic counts for type specimens of *Synodontisvictoriae*. Vertebral counts indicate numbers of total vertebrae: abdominal vertebrae + caudal vertebrae / postanal vertebrae.

Character states	*S.victoriae* BMNH 1906.5.30.191 lectotype	*S.victoriae* BMNH 1906.5.30.190 paralectotype
Standard length (mm)	188.2	225.0
MORPHOMETRIC DATA		
**Percent of standard length**		
Body depth at dorsal fin origin	23.1	25.8
Body depth at anal fin origin	24.1	26.1
Body depth at anal fin insertion	19.0	19.4
Predorsal length	37.3	37.4
Prepectoral length	26.0	25.3
Prepelvic length	54.0	54.4
Preanal length	72.8	77.0
Pectoral–pelvic distance	32.0	32.5
Pelvic–anal distance	20.8	22.9
Caudal-peduncle length	14.7	13.8
Dorsal fin to caudal peduncle	52.4	48.7
Adipose basal length	27.7	31.6
Dorsal-fin depth	25.5	23.9
Anal-fin depth	19.9	18.6
Pectoral-fin length	24.2	24.2
Pelvic-fin length	14.8	15.8
Head length	27.1	27.6
**Percent of head length**		
Head depth at posterior eye margin	57.3	59.0
Head width at posterior eye margin	70.6	68.8
Maximum cranium width	53.7	56.6
Snout length	43.3	48.4
Interorbital width	42.0	43.6
Maxillary-barbel length	97.6	100.3
Outer mandibular-barbel length	50.4	49.8
Inner mandibular-barbel length	32.7	28.8
Humeral-process length	53.1	52.6
Pectoral spine length (unsegmented)	82.2	absent
Dorsal spine length (unsegmented)	81.2	absent
**Percent of snout length**		
Orbit diameter	45.2	37.9
Mouth width	64.3	64.8
Premaxillae width	44.3	32.6
Width of mandibular teeth row	20.8	19.9
**Percent of caudal peduncle length**		
Minimum caudal-peduncle depth	71.7	68.2
Adipose to caudal peduncle	68.5	68.8
**Percent of Dorsal fin to caudal peduncle**		
Dorsal-adipose length	29.3	17.3
MERISTIC DATA		
Dorsal fin rays	II-7	II-8
Anal fin rays	V-9	V-9
Pelvic fin rays	I-6	I-6
Pectoral fin rays	I-9	I-9
Caudal-fin principal rays (upper lobe + lower lobe)	7+8	8+8
Caudal-fin procurrent rays (upper + lower)	12+14	13+13
Mandibular teeth + Primary premaxillary teeth	18+27	20+26
Branches on outer mandibular barbels	4	5
Branches on inner mandibular barbels	5	8
Vertebral counts	39:18+21/19	40:17+23/19

## Supplementary Material

XML Treatment for Synodontis victoriae

## ﻿Additional information

### Author ORCIDs

Gernot K. Englmaier https://orcid.org/0000-0002-9746-4500

Rupert A. Collins https://orcid.org/0000-0002-9135-1169

### Data availability

All of the data that support the findings of this study are available in the main text.
